# The effectiveness of the paclitaxel-coated Luminor® balloon catheter versus an uncoated balloon catheter in superficial femoral and popliteal arteries in preventing vessel restenosis or reocclusion: study protocol for a randomized controlled trial

**DOI:** 10.1186/s13063-016-1657-x

**Published:** 2016-10-28

**Authors:** U. Teichgräber, R. Aschenbach, D. Scheinert, T. Zeller, K. Brechtel, M. Thieme, E. Blessing, M. Treitl, M. Lichtenberg, P. von Flowtow, B. Vogel, M. Werk, V. Riambau, A. Wienke, T. Lehmann, S. Sixt

**Affiliations:** 1 Universitätsklinikum Jena, Institut für Diagnostische und Interventionelle Radiologie, Am Klinikum 1, 07747 Jena, Germany; 2 Universitätsklinikum Leipzig, Abteilung für Interventionelle Angiologie, Philipp-Rosenthal-Straße 27 C, 04103 Leipzig, Germany; 3 Herzzentrum Bad Krozingen, Südring 15, 79189 Bad Krozingen, Germany; 4 Ihre-Radiologen Berlin Gemeinschaftspraxis für Radiologie, Budapester Straße 15-19, 13347 Berlin, Germany; 5 Medinos Kliniken Sonneberg Angiologie/Kardiologie/Diabetologie, Neustadter Str. 61, 96515 Sonneberg, Germany; 6 SRH Klinikum Karlsbad-Langensteinbach, Guttmannstr. 1, 76307 Karlsbad, Germany; 7 Klinikum der Ludwig Maximilians Universität München – Campus Innenstadt, Institut für Klinische Radiologie, Pettenkoferstraße 8a, 80336 München, Germany; 8 Klinikum Arnsberg Angiologie, Stolte Ley 5, 59759 Arnsberg, Germany; 9 Westpfalz-Klinikum GmbH Standort II Kusel, Im Flur 1, 66869 Kusel, Germany; 10 Ruprecht-Karls-Universität Heidelberg, Analysezentrum III/Innere Medizin III, Im Neuenheimer Feld 669, 69120 Heidelberg, Germany; 11 Martin-Luther-Krankenhausbetrieb GmbH, Caspar-Theyß-Straße 27-31, 14193 Berlin, Germany; 12 Hospital Clínic de Barcelona, Carrer de Villarroel, 170, 08036 Barcelona, Spain; 13 Martin-Luther-Universität Halle-Wittenberg, Institut für Medizinische Epidemiologie, Biometrie und Informatik, 06097 Halle (Saale), Germany; 14 Universitätsklinikum Jena, Zentrum für Klinische Studien (ZKS), Postfach, 07740 Jena, Germany; 15 Angiologikum Hamburg, Wördemanns Weg 25-27, 22527 Hamburg, Germany

## Abstract

**Background:**

The aim of this investigator-initiated trial is to evaluate the safety and efficacy of the novel Luminor® paclitaxel-coated drug-eluting balloon (DEB) catheter (iVascular, S.L.U., Barcelona, Spain) in inhibiting restenosis and in ensuring long-term vascular patency.

**Methods/design:**

This is a multicenter randomized controlled trial to evaluate the Luminor® paclitaxel-coated DEB catheter for stenotic or occlusive lesions (length ≤15 cm) in the superficial femoral artery (SFA) and the popliteal artery (PA) up to the P1 segment compared to the noncoated, plain old balloon angioplasty (POBA) catheter. In total 172 subjects will be treated with either the DEB catheter or the POBA catheter in 11 German study centers in a 1:1 randomization study design. The primary endpoint is late lumen loss (LLL) at 6 months. Secondary endpoints are patency rate, target lesion/vessel revascularization, quality of life (assessed with the Walking Impairment Questionnaire (WIQ) and the EQ-5D), change of Rutherford stage and ankle-brachial index, major and minor amputation rate at the index limb, number of dropouts and all-cause mortality.

**Discussion:**

EffPac represents a randomized controlled trial that will provide evidence on the effectiveness of the Luminor® paclitaxel-coated DEB catheter for the reduction of restenosis compared to the POBA catheter for the SFA and the PA. The results of EffPac will allow direct comparison to other already-completed RCTs applying paclitaxel-coated DEBs from different manufacturers with different coating technologies in the same target vessel.

**Trial registration:**

ClinicalTrials.gov Identifier: NCT02540018, registered on 17 August 2015.

Protocol version: CIP Version Final04, 11 February 2016.

EUDAMED No: CIV-15-03-013204.

## Background

The Luminor® paclitaxel-coated drug-eluting balloon (DEB) catheter (iVascular, S.L.U., Barcelona, Spain) is specially designed for the percutaneous transluminal angioplasty (PTA) technique. The DEB catheter is based on a proprietary Transfertech® coating technology. This has been engineered to improve clinical efficacy by optimizing coating properties and device functionalities. It allows a homogeneous and precise paclitaxel concentration of 3 μg/mm^2^ on the PTA balloon surface. This patient-blinded randomized controlled trial (RCT) focuses on the efficacy and the clinical outcome of the Luminor® paclitaxel-coated DEB catheter.

Experience in animal models has shown that paclitaxel-coated DEB catheters demonstrate an inhibition of neointimal hyperplasia after PTA [1]. The safety issue of paclitaxel-coated DEB catheters has been well-investigated in clinical studies for the use in femoropopliteal arteries using paclitaxel-coated DEB catheters from other manufacturers, with drug concentrations ranging from 2.0 to 3.5 μg/mm^2^ on the PTA balloon surface [2]. The THUNDER and FemPac RCTs used the iopromide-matrix-coated Paccocath® catheter with comparable study designs. The results of both trials demonstrated that the paclitaxel-coated DEB catheter offered superior restenosis inhibition and reduced target lesion revascularization (TLR) rates in the femoropopliteal artery [3, 4]. These results were confirmed with IN.PACT Pacific® urea-matrix-paclitaxel-coated DEB catheters in the same target vessel [5].

The EffPac study design follows the Trans-Atlantic Inter-Society Consensus – II (TASC II), which provides comprehensive, evidence-based recommendations for vascular practice and treatment decisions between endovascular and surgical techniques. According to the TASC II on femoropopliteal lesions, endovascular therapy is the treatment of choice for type A lesions (single occlusion ≤5 cm) and is the preferred treatment for type B lesions (occlusions ≤15 cm) (Recommendation No. 37) [6]. The EffPac trial will include both TASC II type A and type B lesions.

### Study hypothesis

That Luminor® paclitaxel-coated balloon catheters will be associated with significantly lower reocclusion and restenosis rates as compared to noncoated, plain old balloon angioplasty (POBA) catheters in both TASC II type A and type B lesions (≤15 cm) of the superficial femoral artery (SFA) and the proximal popliteal artery (PA).

## Methods

### Study design

**Fig. 1:**
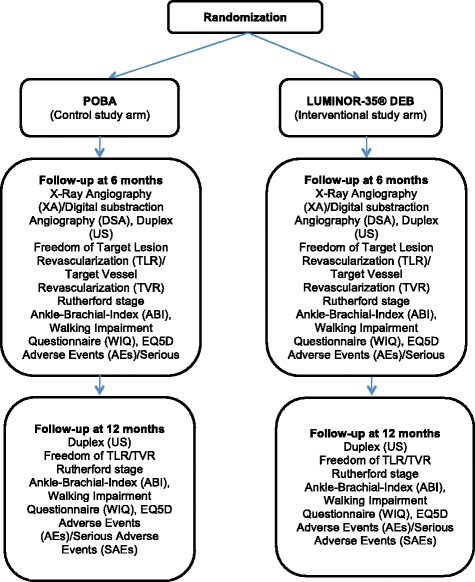
Flow diagram for indication, treatment and follow-up

EffPac is designed as a randomized controlled, multicenter trial in which the Luminor® paclitaxel DEB catheter is compared to the POBA catheter in de novo stenotic/restenotic or occlusive lesions in the SFA and/or proximal PA to prevent vessel restenosis or reocclusion (Fig. 1). The clinical endpoints used for classification focus primarily on effectiveness, followed by safety monitoring and side effects.

### Study setting

The University Hospital Jena, as the coordinating study center, is an academic hospital with intense research activity. The other 10 study sites are located on German national territory. The detailed list of study sites can be accessed at (NCT02540018).

### Primary endpoint

The primary endpoint of EffPac trial is the late lumen loss (LLL), defined angiographically as the difference between the intraluminal vessel diameter (in mm) at 6 months follow-up (FU) and the diameter measured immediately post procedure.

### Secondary endpoints

- Occurrence of restenosis at follow-up (FU) (defined as incidence of stenosis ≥50 %) (6 months and 12 months)
- Freedom from target lesion revascularization (TLR) at FU (6 months and 12 months)
- Freedom from target vessel revascularization (TVR) at FU (6 months and 12 months)
- Change of Rutherford stage at baseline and at FU:
  (0) asymptomatic, (1) mild claudication, (2) moderate claudication, (3) severe claudication, (4) ischemic rest pain, (5) minor tissue loss, (6) ulceration or gangrene
- Change of ankle-brachial Index (ABI) from baseline at FU (6 months and 12 months)
- Change in quality of life according to the WIQ and the EuroQoL 5 dimensions questionnaire (EQ-5D) from baseline at FU (6 months and 12 months)
- Major and minor amputation rate at the index limb
- Number of dropouts
- All-cause mortality

### Definitions

**Fig. 2:**
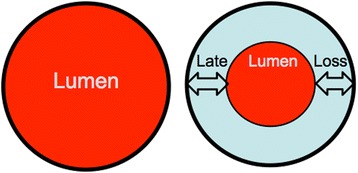
Late lumen loss (LLL) is defined as the angiographic minimum lumen diameter (MLD) immediately after percutaneous transluminal angioplasty (PTA) minus the minimum lumen diameter (MLD) at angiographic follow-up

LLL as the primary efficacy endpoint was not designated as a safety issue, and allows researchers to evaluate the efficacy of paclitaxel-coated DEB angioplasty as a drug device in inhibiting restenosis and reocclusion of target lesions in the SFA. LLL is defined as the angiographic minimum lumen diameter (MLD) immediately after PTA minus the MLD at angiographic follow-up (Fig. 2). The LLL represents a measure that corresponds to neointimal growth inhibition and it predicts TLR occurrence [7]. TLR is defined as a clinically driven repeated percutaneous intervention of the target lesion or bypass surgery of the target vessel.

### Patients

A total of 172 patients (reflecting a 10 % loss to follow-up) with stenosis or occlusion of the SFA and PA, to be randomly assigned to study groups.

### Eligibility criteria

Ages eligible for study: 18 years and older.

Genders eligible for study: both.

Acceptance of healthy volunteers: no.

### Inclusion criteria

The subject must meet all of the following general inclusion criteria:

1. Age ≥18 years
2. Subject must agree to undergo the 6-month angiographic FU as well as the clinical FU (at 6- and 12-months post procedure)
3. Peripheral vascular disease Rutherford classes 2–4
4. De novo stenotic/restenotic lesion or occlusive lesions in the SFA and/or proximal PA
5. If the index lesion is restenotic, the prior PTA must have been more than 30 days prior to treatment in the current study
6. At least 70 % diameter stenosis or occlusion
7. Target lesion length: ≤15 cm (TASC II types A and B)
8. Only one lesion per limb and per patient can be treated
9. At least one patent infrapopliteal run-off artery to the foot of the index limb
10. Successful endoluminal guidewire passage through the target lesion
11. Predilatation prior to randomization
12. Life expectancy, in the investigators’ opinion, of at least 1 year
13. Subject is able to verbally acknowledge and understand the aim of this trial and is willing and able to provide informed consent

### Exclusion criteria

The subject must not meet any of the following general exclusion criteria:

1. Previous surgery in the target vessel
2. Patients who require a PTA balloon catheter of diameter size 4 mm and below or diameter size above 7 mm
3. Major amputation in the same limb as the target lesion
4. Acute myocardial infarction within 30 days before intervention
5. Severely calcified target lesions in the SFA/PA resistant to PTA
6. Subjects requiring different treatment or raising serious safety concerns regarding the procedure or the required medication
7. Women of childbearing potential, except women meeting the following criteria:
  Post-menopausal (12-month natural amenorrhea or 6-month amenorrhea with serum FSH >40 mlU/ml)
  Sterilization 86 weeks after bilateral ovariectomy with or without hysterectomy
  Using an effective method of birth control for the duration of the trial: implants, injectables, combined oral contraceptives, intrauterine device (in place for a period of at least 2 months prior to screening) and with a negative serum pregnancy test
  Sexual abstinence
  Vasectomized partner
8. Pregnant and nursing women
9. Acute thrombus or aneurysm in the index limb or vessel (presence of stent in the target lesion)
10. In-stent restenosis in the target lesion
11. Renal insufficiency with a serum creatinine >2.0 mg/dl at baseline
12. Platelet count <50 G/l or >600 G/l at baseline
13. Known hypersensitivity or contraindication to contrast agent that cannot be adequately premedicated
14. Subjects with known allergy to paclitaxel
15. Subjects with intolerance of antiplatelet, anticoagulant, or thrombolytic medications that would be administered during the trial
16. Dialysis or long-term immunosuppressant therapy
17. Current participation (or within the last 3 months) in another interventional study
18. Treatment of in-stent restenosis of target lesion is not allowed, but treatment of in-stent restenosis outside of target lesion in the target vessel is not an exclusion criterion

### Recruitment

All compliant patients undergoing angioplasty to the SFA and PA are accurately informed about the trial in a prescreening phase. The study nurse visits the eligible patient on the ward or at the outpatient clinic and explains to them the objectives and procedures that are relevant to the study and hands them information material such as the study flyer. If the patient gives consent to participate in the EffPac trial, the investigator is responsible for obtaining written informed consent from the patient after adequate explanation of all aspects of the trial that are relevant for the patients’ decision to participate. Before participation in the study, the investigator must also inform the patients that they are completely free to refuse to enter the study or to withdraw from it at any time and for any reason. The patient is enrolled in the study after it has been determined that they meet all the inclusion criteria and none of the exclusion criteria. The investigator enrolls the patients and assigns the participants to intervention.

### Safety and quality control

A Data and Safety Monitoring Board (DSMB) is established in order to protect the safety, rights and wellbeing of study participants. The DSMB consists of a group of two independent physicians and a statistician with pertinent expertise who review and adjudicate important endpoints and relevant adverse events reported by the study investigators. The DSMB members serve as an advisory panel to the study investigators.

Compensation for post-trial care, for those patients who suffered harm from trial participation, is covered by a trial health insurance.

### Confidentiality

The investigators and the study staff will keep all information provided by the sponsor and about the study patients in strict confidence. For protection of these data, organizational procedures are implemented to prevent their distribution to unauthorized persons. Appropriate local data legislation will be fulfilled in its entirety.

### Financial disclosure

All investigators must sign the study protocol acceptance and the financial disclosure form.

### Medications

Patients will receive dual antiplatelet therapy (DAPT) during a period of at least 4–6 weeks after angioplasty, if tolerated. The typical, recommended DAPT regimen consists of acetylsalicylic acid (ASA) (minimum 75 mg/day) and a P2Y12 inhibitor, e.g., clopidogrel (minimum 75 mg/day). Alternate DAPT regimens may be followed if justified by individual patient requirements, e.g., if there is documented intolerance to any of these drugs, or the patient is already taking a different antiplatelet therapy (with at least two approved drugs) due to comorbid conditions. The investigator will be guided by the drug manufacturer’s instructions, available scientific evidence and medical guidelines applicable to patients with peripheral arterial disease.

After 4–6 weeks of DAPT post procedure, patients will receive at least one antiplatelet drug indefinitely. Patients receiving anticoagulant therapy should not receive additional antiplatelet drugs if, in the opinion of the investigator, this could present an intolerable bleeding risk.

Patients who do not take already DAPT drugs at admission should receive an appropriate periprocedural loading doses. Recommended minimum loading doses are 300 mg for ASA and 300 mg for clopidogrel. At least one antiplatelet drug is administered prior to the angioplasty, and the second antiplatelet drug should be administered within 2 h post procedure if not given beforehand. At the time of the procedure, patients should receive an intra-arterial bolus of heparin (usually 5000 IU), or alternate anticoagulants as substitutes for heparin if justified by individual patient requirements. The typical, recommended DAPT and anticoagulation regimen must be consistently followed for both study and control devices.

### Adverse and serious events

An adverse event (AE) is any untoward medical occurrence in a subject that occurs in conjunction with the use of the study device, whether or not the event is considered device-related.

New and worsening signs and symptoms of underlying or emerging disease must be recorded as adverse events.

Adverse events may include, but are not limited to:

- Subjective or objective symptoms spontaneously reported by the patient or subject and/or observed by the investigator or medical staff
- Laboratory, electrocardiogram (ECG) or physical examination abnormalities of clinical significance or for which a medical intervention was initiated

A serious adverse event (SAE) or serious adverse device effect (SADE) is defined as an AE or adverse device effect (ADE) that results in any of the following outcomes:

- Death,
- A life-threatening adverse event,
- Inpatient hospitalization or prolongation of existing hospitalization,
- A persistent or significant disability/incapacity
- A congenital anomaly/birth defect

Important medical events that may not result in death, be life-threatening, or require hospitalization may be considered a SAE when, based upon appropriate medical judgment, they may jeopardize the subject and may require medical or surgical intervention to prevent one of the outcomes listed in the definition above.

All AEs during the safety observation period, whether considered associated with the use of the study endovascular device or not, must be monitored until symptoms subside or return to baseline, or until there is a satisfactory explanation for the changes observed. Follow-up information will be submitted to the study sponsor as it becomes available.

The safety observation period starts with successful randomization and ends at the 12-month follow-up (visit 3).

### Statistical analysis

All analyses of primary and secondary endpoints follow the intention-to-treat (ITT) principle and are performed for all participants based on the group to which they were randomly allocated. Multiple imputation of missing values will be conducted for the primary endpoint to evaluate the robustness of the conclusions.

### Sample size

The primary objective of the trial is to show that LLL in the paclitaxel-coated DEB catheter group is less than that in the POBA catheter group.

In a pilot trial by Werk et al. [3], average lumen loss after 6 months was 0.5 mm (SD = 1.1 mm) in the coated balloon group and 1.0 mm (SD = 1.1 mm) in the uncoated balloon group. At a 5 % significance level, a two-sided independent samples *t* test will have an 80 % power to detect an effect size of 0.45 when the sample size in each group is 77 (nQuery Advisor 7.0). Taking into account a dropout rate for primary endpoint data of 10 %, a total of 172 patients should be randomized.

### Data analysis plan

The primary endpoint will be analyzed by a linear mixed model with fixed effect of the treatment and random center effects.

Kaplan-Meier curves will be estimated for the secondary endpoints of mortality and minor and major amputations. The survival curves of the treatment groups are compared using the log-rank test.

For Rutherford stage, the change in class number between baseline and follow-up will be calculated for each patient. To compare both treatment groups regarding the change, Cochran-Mantel-Haenszel statistics will be applied.

Change of ABI and “Quality of Life” according to the EQ-5D and the WIQ will be compared between the treatment groups by an independent sample *t* test or a nonparametric Mann-Whitney *U* test according to the data distribution. Frequency of restenosis, number of dropouts, TLR and TVR will be compared by Fisher’s exact test between groups.

### Study schedule

#### Visit 0: (baseline period/screening)

The screening process will be used to determine the inclusion or exclusion of a patient in the study. After the patient has signed the informed consent form, the screening process may begin.

This process includes the investigator’s assessment of the patient’s medical records and diagnosis, including:

- Demographic data: gender, date of birth, height, weight, smoking status
- Medical history: (including concomitant diseases (hypertension, hyperlipidemia, diabetes mellitus, renal insufficiency, angina pectoris, arrhythmia, stroke, congestive heart failure, coronary arterial disease, transient ischemic attack, myocardial infarction)
- Concomitant medication and medical treatments
- Anticoagulants
- Physical assessment
- Routine laboratory tests (with pregnancy test in women with childbearing potential)
- Duplex ultrasound (DUS) of the pelvic and lower-limb arteries – optional (according to the physician’s decision)
- EQ-5D and Walking Impairment Questionnaire (WIQ)
- Ankle-brachial Index (ABI)
- Rutherford stage assessment

#### Visit 1: treatment (angioplasty)

The balloon dilatation procedure, including deployment to the target lesion and balloon inflation, deflation and retrieval, is performed under fluoroscopic observation. All sites will have access to an emergency unit to enable interventions to be converted to bypass surgery, e.g., in case of failed PTA. The patient is positioned on the angiographic table and draped in a sterile fashion. The standard vascular access accords with the ipsilateral or contralateral femoral artery in relation to the target vessel. The endovascular procedure can be performed in a direct antegrade or a crossover retrograde technique.

An introducer sheath will be inserted over a guidewire. Five thousand I.U. heparin is injected intra-arterially (i.a.) to prevent periprocedural thrombotic events. Alternative periprocedural anticoagulation regimens may be applied if justified by individual patient requirements. Endoluminal guidewire passage through the stenotic and occlusive femoropopliteal lesion is mandatory for study inclusion.

A POBA PTA balloon of appropriate balloon diameter and length and catheter working length is selected according to the characteristics of the target vessel and the lesion for predilatation and assessment by angiography (digital subtraction angiography (DSA) or X-ray angiography (XA)). A ruler must be placed adjacent to the target vessel for accurate measuring.

Predilatation in both study arms with POBA catheters:

- Inflation time minimum 30 s (without limit above)
- PTA balloon inflation up to nominal pressure
- Diameter of POBA catheter according to reference measurement directly proximal to the lesion
- Length of POBA catheter with 10 mm overlap proximal and distal to the lesion
- Repeated predilatations (up to three) with the same POBA catheter is allowed in case of PTA-resistant stenosis

After predilatation of the target lesion, an angiographic assessment will be performed (DSA or XA). Again, a ruler must be placed adjacent to the target vessel for accurate measuring. The treatment group represents the Luminor® DEB catheter and the control group the POBA catheter using a CE-marked non-drug-eluting PTA balloon catheter.

Randomization occurs only after the target lesion has been successfully crossed by a guidewire and there is fulfillment of angiographic inclusion/exclusion criteria. Randomization will be performed by sealed envelope pull. Due to the nature of the study, the investigator will not be blinded to the treatment allocation. The allocation is patient-blinded. Unblinding is only permissible if knowledge of the study treatment is absolutely essential for further management of the patient.

For both Luminor 35® paclitaxel-coated DEB angioplasty (experimental group) and the POBA catheter (control group), identical procedures for both study arms will be adopted:

- Inflation time 60 ± 10 s
- PTA balloon inflation up to nominal pressure
- Diameter of PTA balloon catheter should be sized ≥1:1 according to reference vessel diameter measurement directly proximal to the lesion
- Length of PTA catheter with 10 mm overlap proximal and distal to the lesion
- The longest possible adaptable balloon must be used for the lesion. If this is not applicable, overlapping is allowed
- In case two or multiple DEB catheters are used a minimal overlap of 5 to 10 mm is required

After dilatation of the target lesion, the PTA catheter is withdrawn through the introducer sheath and a post-PTA angiogram is performed (DSA or XA) to evaluate the technical result and possible procedural complications. A final run-off angiogram (DSA or XA) of the BTK (below the knee) arteries is required.

**Fig. 3:**
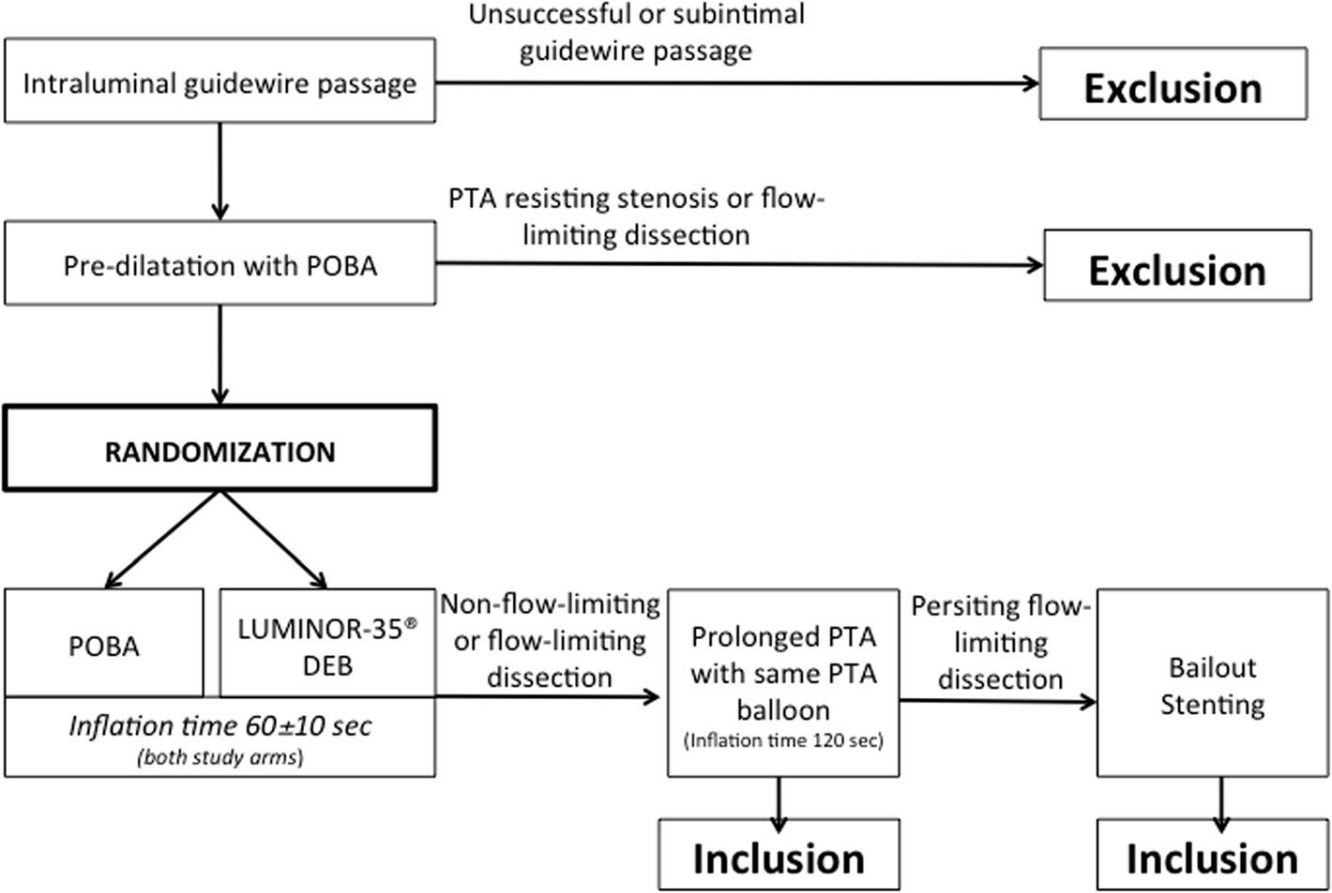
Randomization and angioplasty

In case of a nonflow-limiting or flow-limiting dissection, a prolonged PTA with the same PTA balloon is required. If the flow-limiting dissection persists, the required dropout stenting is not an exclusion criterion (Fig. 3).

If stenting is necessary, stent grafts (covered stents) or drug-coated stents (heparin, paclitaxel, etc.) cannot be used.

#### Visit 2: 1. Follow-up (6-month follow-up (±30 days))

The 6-month follow-up is an in-person visit and the following data will be collected:

- Angiographic assessment/quantitative vascular analysis (QVA) (primary endpoint: LLL) (if further treatment becomes necessary, drug-eluting stent (DES) or DEBs should not be used, only POBA catheters)
- DUS to identify the occurrence of restenosis
- Medication
- TLR/TVR
- ABI
- Rutherford stages
- WIQ, EQ-5D
- AEs/SAEs

#### Visit 3: 2. Follow-up (12-month follow-up (±30 days))

The 12-month follow-up is an in-person visit and the following data will be collected:

- DUS to identify the occurrence of restenosis
- Medication
- TLR/TVR
- ABI
- Rutherford stages
- WIQ, EQ-5D
- AEs/SAEs

To promote follow-up completion, the study nurse is in continuous contact with the study patients and in timely fashion sends letters to invite patients to the follow-up visits.

A schedule of enrollment, interventions and assessments for the EffPac trial following Standard Protocol Items: Recommendations for Interventional Trials (SPIRIT) is summarized in Table 1.

**Table 1:** Schedule of enrollment, interventions and assessments for the EffPac trial

	Study period					
	Enrollment	Allocation	Post allocation			Close-out
Time point^a^	*−t*_*1*_	0	*t*_*1*_	*t*_*2*_	*t*_*3*_	*t*_*x*_
Enrollment:						
Prescreening	X					
Eligibility screen	X					
Informed consent	X					
Allocation		X				
Interventions:						
Intervention DEB catheter			X			
Control group POBA catheter			X			
Assessments^b^:						
List baseline variables: XA/DSA				X	X	X
Duplex ultrasound (DUS)				X	X	X
Freedom from TLR/TVR				X	X	X
Rutherford stage				X	X	X
ABI				X	X	X
WIQ				X	X	X
EQ-5D				X	X	X
AE				X	X	X
SAE				X	X	X
Primary endpoint: LLL				X		
Secondary endpoints: Occurrence of restenosis				X	X	X
Freedom of TLR				X	X	X
Freedom of TVR				X	X	X
Change of Rutherford				X	X	X
Change of ABI				X	X	X
Patency (DUS)				X	X	X
Change of QoL (WIQ and EQ-5D)				X	X	X
Amputation rate				X	X	X
Dropouts				X	X	X
Mortality				X	X	X

^a−^ *t*_*1*_ screening and enrollment, *t*_*1*_ baseline (visit 0), *t*_*2*_ follow-up 1 after 6 months (visit 1), *t*_*3*_ follow-up 2 after 12 months (visit 2), *t*_*x*_ close-out = end of follow-up 2

^b^ *ABI* Ankle-brachial Index, *AE* adverse events, *DEB* drug-eluting balloon, *DSA* digital subtraction angiography, *LLL* late lumen loss, *POBA* plain old angioplasty balloon, *QoL* quality of life, *SAE* serious adverse events, *TLR* target lesion revascularization, *TVR* target vessel revascularization, *US* ultrasound, *WIQ* Walking Impairment Test, *XA* X-ray angiography

### Monitoring and reporting

The sponsor or the appointed Clinical Research Organization (CRO) is responsible for the monitoring activities. The site initiation, monitoring and close-out visits in general will be performed on site. The purpose of the monitoring is primarily to ensure the protection of the rights and safety of the participating subjects. After the monitoring visits a detailed report will be created. Protocol deviations that occur, such as AEs and SAEs, are discussed with the investigator and reported to the sponsor within 24 h. Furthermore, the monitoring is to ensure good data quality and a continuing conduction of the clinical trial in accordance with the CIP, ISO 14155, the ICH-GCP and the Declaration of Helsinki.

### Data collection

For each randomized patient an electronic Case Report Form (CRF) will be completed. Data capture takes place via web application on the servers of the Center for Clinical Studies at Jena University Hospital with “OpenClinica®”, a study management software. OpenClinica® meets all regulatory requirements (GCP, 21CFR Part 11).

Patient files and other source data (particularly with regard to informed consent, date of X-rays and outcome) must be kept for the maximum period of time permitted by the hospital or institution, but at least 30 years. The sponsor of the trial must keep all other documentation pertaining to the trial for at least 15 years.

### Auditing

All local and national regulations are observed. This includes an audit by the sponsor’s representatives during the course of the study. All protocol modifications were communicated to the relevant parties.

### Amendment

In February 2016 an amendment was implemented after notification and approval by the regulatory authorities.

## Trial status

Recruiting.

Estimated enrollment: 172 including estimated 10 % loss of follow-up.

Study start date: August 2015.

Study completion date: December 2017.

## Sponsor

Universitätsklinikum Jena, Bachstrasse 18, 07745 Jena, Germany.

## Discussion

Multiple randomized “first-in-man” trials and registries [3, 4, 8, 9] of first-generation drug-coated balloon technology have convincingly demonstrated the superiority of paclitaxel-coated DEB angioplasty compared to POBA in relation to LLL, restenosis rate and freedom from TLR. In line, a meta-analysis including 381 patients overall (DEB angioplasty *N* = 186 versus POBA *N* = 195) confirmed angiographically the superiority of paclitaxel-coated DEB angioplasty in reducing significantly the TLR rate (12.2 % versus 27.7 %, respectively) and the restenosis rate (18.7 % versus 45.5 %, respectively) compared to POBA [10]. Interestingly, up to now only drug-eluting devices for peripheral interventions based on paclitaxel-coated technology have demonstrated clinical benefit. There are nine RCTs comparing paclitaxel-coated DEB angioplasty versus POBA, five of them industry-sponsored. A total of 1448 patients were recruited (range, 50 to 479). The most common primary endpoint is “late lumen loss” (LLL) at 6 months. Seven out of nine RCTs were core-laboratory adjudicated for primary outcome assessment (Table 2) [3–5, 8, 11–17]. First, paclitaxel (3 μg/m^2^) was combined with iopromide as an excipient (Paccocath) [3, 4] followed by others such as polysorbate and sorbitol (Lutonix 35, LEVANT I) [8] or urea (IN.PACT Pacific, Pacifier®) [5] and butyryl-trihexyl citrate carrier substances (Passeo Lux, Biolux P-1) [11].

**Table 2:** Study overview of published RCTs applying paclitaxel-coated DEB angioplasty in the femoral and popliteal arteries

Study	Author/Journal/Year	Patients	Study device	Primary endpoint	Sponsor
BIOLUX P-I [11]	Scheinert D, JET 2015	60	Biotronik Passeo-18 LUX	LLL 6 mo	Biotronik
DEBATE-SFA [12]	Liistro F, JACC 2013	104	Medtronic IN.PACT Admiral	binary restenosis 12 mo	Independent
DEBELLUM [13]	Fanelli F, JCV 2014	50	Medtronic IN.PACT Admiral or IN.PACT Amphirion	LLL 6 mo	Independent
FemPac [3]	Werk M, CIRC 2008	117	PACCOCATH	LLL 6 mo	Independent
IN.PACT SFA [14, 15]	Tepe G, CIRC 2015; Laird J, JACC 2015	331	Medtronic IN.PACT Admiral	pP 12 mo	Medtronic
LEVANT 1 [8]	Scheinert D, JACC 2014	101	Bard Lutonix DCB	LLL 6 mo	Bard
LEVANT 2 [16]	Rosenfield K, NEJM 2015	476	Bard Lutonix DCB	pP 12 mo	Bard
PACIFIER [5]	Werk M, CIRC 2012	85	Medtronic IN.PACT Admiral	LLL 6 mo	Independent
THUNDER [4, 17]	Tepe G, NEJM 2008, JACC 2015	154	PACCOCATH	LLL 6 mo	Bavaria Medizintechnologie, Schering

*LLL* late lumen loss, *mo* months, *pP* primary patency

After having demonstrated the proof of concept, two RCTs followed: the IN.PACT SFA trial [12], using the IN.PACT Admiral® (Medtronic, Santa Rosa, CA, USA) balloon catheter (urea and 3.5 μg/m^2^ paclitaxel) and enrolling 331 patients revealed at 12 months a significantly lower clinically driven TLR rate for DEB angioplasty compared to uncoated POBA (2.4 % versus 20.6 %; *p* < 0.001) and a higher primary patency rate of 82.2 % for DEB angioplasty versus 52.4 % for POBA; the second trial (LEVANT II) [16] included 476 patients overall who were treated with the Lutonix® (Bard, Covington, GA, USA) paclitaxel-coated DEB angioplasty (polysorbate/sorbitol and 2 μg/m^2^ paclitaxel). The primary patency at 12 months was favorable for paclitaxel-coated DEB angioplasty (65.2 % versus 52.6 %; *p* = 0.02) and again demonstrated a better outcome. However, at 12 months freedom from clinically driven TLR in the LEVANT II trial was not significantly improved compared to POBA (87.7 % versus 83.2 %; *p* = 0.208). Again, both trials confirmed that balloon-based paclitaxel drug-eluting technology for treatment of TASC II types A and B femoropopliteal artery lesions achieve promising clinical 1-year outcomes. Consistently, no signs of paclitaxel-coated DEB angioplasty-associated side effects, including amputation or distal embolization of the vascular bed, were reported during follow-up in any of the studies. In line, the Stellarex® paclitaxel-coated DEB catheter (Spectranetics, Colorado Springs, CO, USA) was also shown to be safe and effective with a lower paclitaxel concentration (2 μg/m^2^ paclitaxel) according to recent 24-month data released from the first in-man study (ILLUMINATE) [18]. In this prospective, multicenter, single-arm study 58 femoropopliteal artery lesions in 50 patients were treated and the preliminary data revealed a primary patency rate of 80.3 % and freedom from clinically driven TLR of 85.8 % with no deaths or amputations at 24 months. Data from already-initiated, randomized multicenter trials comparing Stellarex® paclitaxel-coated DEB angioplasty versus POBA are, however, still ongoing.

As uncompromised drug delivery to the target lesion in the vasculature is a major issue, strong efforts were made to address this goal. For example, Boston Scientific (Marlborough, MA, USA) has investigated the Ranger® paclitaxel-coated DEB catheter. This device has a proprietary trans-Pax coating® technology designed to maintain drug-coating integrity and maximize drug transfer efficiency. All the different paclitaxel-coated DEB catheters available until now have in common that their proprietary coating technology enables consistent and predictable drug delivery to the vessel wall.

As mentioned above, the investigators of this trial evaluated the concept of homogenous distribution of paclitaxel 3 μg/cm^2^ on the balloon’s surface by applying a proprietary Transfertech® coating technology. The drug is deposited on the balloon’s surface by means of ultrasonographic exposure to guarantee a uniform diameter in nanodrops to finally constitute an ultrathin multilayer coating. This technology has been engineered to improve clinical efficacy by optimizing coating properties and device functionalities. In animal models, this concept showed convincing results and promising first in-men studies resulted in the CE mark being granted for the Luminor® DEB catheter.

Overall, paclitaxel-coated DEB angioplasty is an innovative technology that offers multiple advantages and follows a “leaving-nothing-behind” concept that maintains all future options open for further treatment. This possibility fits well, as the natural course of peripheral arterial disease is, unfortunately, progressive.

## Abbreviations

ABI: Ankle-brachial Index
ADE: Adverse device effect
AE: Adverse event
BTK: Below the knee
CRF/eCRF: Case Report Form/electronic Case Report Form
DAPT: Dual antiplatelet therapy
DCB: Drug-coated balloon
DEB: Drug-eluting balloon
DMSB: Data Monitoring and Safety Board
DSA: Digital subtraction angiography
DUS: Duplex ultrasound
FU: Follow-up
ITT: Intention-to-treat
LLL: Late lumen loss
MLD: Minimum lumen diameter
PA: Popliteal artery
PI: Principal investigator
POBA: Plain old balloon angioplasty
PTA: Percutaneous transluminal angioplasty
RCT: Randomized controlled trial
SAE: Serious adverse event
SFA: Superficial femoral artery
TASC: Trans-Atlantic Inter-Society Consensus
TLR: Target lesion revascularization
TVR: Target vessel revascularization
US: Ultrasound
WIQ: Walking Impairment Questionnaire
XA: X-ray angiography
