# Erratum to: The effectiveness of the paclitaxel-coated Luminor® balloon catheter versus an uncoated balloon catheter in superficial femoral and popliteal arteries in preventing vessel restenosis or reocclusion: study protocol for a randomized controlled trial

**DOI:** 10.1186/s13063-017-1884-9

**Published:** 2017-04-26

**Authors:** U. Teichgräber, R. Aschenbach, D. Scheinert, T. Zeller, K. Brechtel, M. Thieme, E. Blessing, M. Treitl, M. Lichtenberg, P. von Flotow, B. Vogel, M. Werk, V. Riambau, A. Wienke, T. Lehmann, S. Sixt

**Affiliations:** 1 Institut für Diagnostische und Interventionelle Radiologie, Universitätsklinikum Jena, Am Klinikum 1, 07747 Jena, Germany; 2 Abteilung für Interventionelle Angiologie, Universitätsklinikum Leipzig, Philipp-Rosenthal-Straße 27 C, 04103 Leipzig, Germany; 3 Herzzentrum Bad Krozingen, Südring 15, 79189 Bad Krozingen, Germany; 4 Ihre-Radiologen Berlin Gemeinschaftspraxis für Radiologie, Budapester Straße 15-19, 13347 Berlin, Germany; 5 Medinos Kliniken Sonneberg Angiologie/Kardiologie/Diabetologie, Neustadter Str. 61, 96515 Sonneberg, Germany; 6 SRH Klinikum Karlsbad-Langensteinbach, Guttmannstr. 1, 76307 Karlsbad, Germany; 7 Klinikum der Ludwig Maximilians, Universität München – Campus Innenstadt, Institut für Klinische Radiologie, Pettenkoferstraße 8a, 80336 München, Germany; 8 Klinikum Arnsberg Angiologie, Stolte Ley 5, 59759 Arnsberg, Germany; 9 Westpfalz-Klinikum GmbH Standort II Kusel, Im Flur 1, 66869 Kusel, Germany; 10 Analysezentrum III/Innere Medizin III, Ruprecht-Karls-Universität Heidelberg, Im Neuenheimer Feld 669, 69120 Heidelberg, Germany; 11 Martin-Luther-Krankenhausbetrieb GmbH, Caspar-Theyß-Straße 27-31, 14193 Berlin, Germany; 12 Hospital Clínic de Barcelona, Carrer de Villarroel, 170, 08036 Barcelona, Spain; 13 Institut für Medizinische Epidemiologie, Biometrie und Informatik, Martin-Luther-Universität Halle-Wittenberg, 06097 Halle (Saale), Germany; 14 Zentrum für Klinische Studien (ZKS), Universitätsklinikum Jena, Postfach, 07740 Jena, Germany; 15 Angiologikum Hamburg, Wördemanns Weg 25-27, 22527 Hamburg, Germany

## Erratum

In the original publication [1] the spelling of one author name was incorrect. The correct version: Peter von Flotow.
